# P-Glycoprotein Aggravates Blood Brain Barrier Dysfunction in Experimental Ischemic Stroke by Inhibiting Endothelial Autophagy

**DOI:** 10.14336/AD.2022.0225

**Published:** 2022-10-01

**Authors:** Liangliang Huang, Yan Chen, Rui Liu, Binbin Li, Xuan Fei, Xiang Li, Ge Liu, Yunman Li, Baohui Xu, Weirong Fang

**Affiliations:** ^1^State Key Laboratory of Natural Medicines, School of Basic Medical Sciences and Clinical Pharmacy, China Pharmaceutical University, Nanjing, Jiangsu 210009, China.; ^2^Department of Surgery, Stanford University School of Medicine, Stanford, CA 94305, USA

**Keywords:** P-glycoprotein, ischemic stroke, blood brain barrier, tight junction, autophagy

## Abstract

P-glycoprotein (P-gp) is expressed on brain microvessel endothelial cells of blood-brain barrier (BBB) and elevated after cerebral ischemia. In this study, we explored the influence and potential mechanisms of P-gp on BBB function in experimental ischemic stroke *in vivo* and *in vitro*. Middle cerebral artery occlusion/reperfusion (MCAO/R) was created in mice. Oxygen-glucose deprivation/reoxygenation (OGD/R) was performed in brain microvascular vessel-derived endothelial cells (bEnd.3) to mimic ischemia/reperfusion injury *in vitro*. P-gp-specific siRNA and pharmacological inhibitor cyclosporine A were used to inhibit P-gp, whereas pcDNA3.1 was utilized to overexpress P-gp. Twenty-four hours after reperfusion, acute ischemic stroke outcome, BBB integrity and permeability, autophagic proteins and relative signaling pathways were evaluated. P-gp levels were markedly elevated in mouse brain and endothelial cells following MCAO/R and OGD/R, respectively. P-gp siRNA silencing or pharmacologically inhibiting (cyclosporine A) reduced infarct volume and brain edema, attenuated brain pathology, and improved neurological behavior in association with attenuated accumulation of neutrophils and macrophages, reduced expression levels of inflammatory cytokines (TNF-α and IL-1β), matrix metalloproteinases (MMP-2 and MMP-9) and adhesion molecules (ICAM-1 and VCAM-1). P-gp silence also counteracted BBB leakage, restored the expressions of tight junction proteins (Claudin-5, Occludin and ZO-1), activated autophagic proteins (upregulated LC3-II/LC3-I and Beclin 1, and downregulated P62), and diminished Akt/mTOR signal activity in mice following MCAO/R. In the endothelial cell OGD/R assay, P-gp silence downregulated the expressions of inflammatory cytokines and adhesion molecules, inhibited leukocytes adhesion and migration, increased tight junction protein levels, and activated autophagy, all were reversible by forceful P-gp expression. Additionally, treatment with an autophagy inhibitor (3-methyladenine) abolished protections against ischemic stroke and tight junction proteins reduction followed by P-gp silence. In conclusion, increased P-gp expression after ischemic injury resulted in BBB dysfunction and hyperpermeability by suppressing Akt/mTOR-induced endothelial autophagy.

Stroke is one of leading causes of disability and mortality worldwide, with ischemic stroke accounting for 87% of all stroke-related incidents [1]. Inflammatory cascade is activated followed by secondary inflammatory responses beginning within minutes and persisting for days or even weeks after ischemic stroke. Endothelial cells produce matrix metalloproteinases (MMPs) and inflammatory factors to destroy extracellular matrix and thus disrupt blood-brain barrier (BBB) integrity [2, 3]. Furthermore, inflammatory factors such as tumor necrosis factor-α (TNF-α) and interleukin-1β (IL-1β) also inhibit tight junction proteins (TJPs) expression, leading to BBB hyperpermeability [4, 5]. In the mouse model of cerebral ischemia/reperfusion, BBB leakage occurs 30 min after reperfusion and increases the risk for intracerebral hemorrhage prior to infarction formation [6]. Therefore, maintaining BBB integrity reduces cerebral ischemic injury.

P-glycoprotein (P-gp), also known as multidrug resistance transporter-1, is a membrane transport protein belonging to adenosine triphosphate (ATP)-binding cassette (ABC) transporter superfamily [7]. As a major efflux transporter, P-gp is predominantly expressed on the luminal site of brain microvessel endothelial cells (BMVECs) that form BBB [8]. P-gp is well known for maintaining brain homeostasis by pumping drugs out of brain [9]. In recent studies, altered P-gp expression is associated with pathogenesis of several neurological diseases including multiple sclerosis, Alzheimer's disease, and Huntington’s disease [10-12]. P-gp is also increased on capillary endothelia after focal cerebral ischemia [13, 14]. However, the specific role and underlying mechanisms of P-gp on BBB function and integrity remain largely unknown.

Autophagy is a self-eating cellular process to maintain cellular homeostasis and normal cellular functions [15], and is activated in various cells such as neurons, glia cells, and BMVECs in the brain [16]. Endothelial autophagy activation following cerebral ischemia restores and maintains BBB integrity that attenuate brain edema and protect against stroke [17, 18]. Conversely, treatment with an autophagy inhibitor (3-methyladenine, 3-MA) induced cell apoptosis both in oxygen-glucose deprivation/reoxygenation (OGD/R)-injured BMVECs and ischemic rats [19]. It has been reported that canagliflozin inhibited P-gp function and autophagy in HepG2 cells and improved the sensitivity to the antitumor effect of doxorubicin [20]. However, it has not been investigated whether increased P-gp influences endothelial autophagy in ischemic stroke.

In this study, we investigated the effect and its underlying mechanisms of P-gp on BBB integrity in the mouse model of ischemic stroke. We found that elevated microvascular endothelial P-gp degraded endothelial tight junction, increased BBB hyperpermeability, accelerated brain inflammation and thus worsened ischemic stroke outcomes in association with increased Akt/mTOR activity and reduced endothelial autophagy.

## MATERIALS AND METHODS

### Animals

Male C57BL/6 mice weighting 22-25 g were purchased from Qinglongshan Animal Breeding Centre (Nangjing, Jiangsu, China) and used in all experiments. All animals were housed and fed freely in a temperature-controlled room (22 ± 2°C) with a 12 h light-dark cycle. All experimental procedures were performed under licenses that granted by China Pharmaceutical University Animal Experimental committee (Nanjing, Jiangsu, China), in compliance with the National Institutes of Health (Bethesda, MD, USA) guidelines for the care and use of animals. Mice were randomly assigned into 6 treatments, including sham treatment, middle cerebral artery occlusion/reperfusion (MCAO/R) treatment, MCAO/R with negative control siRNA (NC siRNA) treatment, MCAO/R with NC siRNA and cyclosporine A treatments (NC siRNA + CsA), MCAO/R with P-gp siRNA treatment (MCAO/R + P-gp siRNA), MCAO/R with P-gp siRNA and 3-MA treatments (MCAO/R + P-gp siRNA + 3-MA). The treatment was administered in a blinded fashion. The researcher conducting the surgeries and behavior tests was unaware of the animal groups, and the researcher analyzing the data did not know the group condition.

### Intracerebroventricular (i.c.v) injection and drug administration

Mice were anesthetized by 3% isoflurane and maintained using 1.5% isoflurane (R540S, RWD, Shenzhen, Guangdong, China) and placed in a stereotaxic frame (Chengdu Technology & Market, Chengdu, Sichuan, China) as previously described [21]. The skin was cut open along the head midline, and a small burr hole (0.5 mm diameter) was drilled into the skull (0.3 mm posterior to bregma; 1.0 mm lateral to sagittal suture). P-gp (abcb1A, GGAUCCAGUCUAAUAAGAATT) siRNA or its NC siRNA (1.5 μL/10 g body weight, 1 nmol/μL, KeyGEN BioTECH, Nanjing, Jiangsu, China) was intracerebroventricularly injected into right lateral ventricle (depth: 2.5 mm dorsal) within 4 min period. siRNA knockdown efficiency was assayed to optimize the time of P-gp siRNA administration by Western blot. The results are shown in Supplementary Fig 1. Assham control, mice were injected with same volume of saline intracerebroventricularly. The wound was closed, and mice were recovered on a heating pad.

Autophagy inhibitor (3-MA) and P-gp inhibitor (CsA) were purchased from MedChemExpress LLC (Monmouth Junction, NJ, USA). 3-MA was prepared in normal saline immediately before use, and intracerebroventricularly injected at a dose of 15 μg per mouse 30 min prior to MCAO surgery [22]. CsA was dissolved in DMSO (40 mg/mL), diluted with saline prior to use, and given at the dose of 22 mg/kg body weight/intravenous immediately after the MCAO surgery [23].

### Focal cerebral ischemia

Following 12 h fasting, MCAO/R was performed as described previously [24]. Mice were subjected to anesthesia, and cerebral blood flow (CBF) was monitored via Laser Speckle Contrast Imager (Moor Instruments, Essex, UK). The result is provided in Supplementary Fig. 2. Right common carotid artery (CCA) was carefully dissected, and right external carotid artery (ECA) was then separated, and then the right internal carotid artery (ICA) was isolated. Silicon-coated monofilament nylon suture (external diameter 0.16 mm) was slowly inserted into ICA from ECA gently and advanced about 11 mm to block the blood supply to MCA. The filament was left for 90-min obstruction and then withdrawn for the reperfusion. The sham group was operated identically, except for no MCA occlusion. Exclusion criteria are as follows: 1) obvious bleeding during the operation; 2) CBF following MCAO surgery is more than 30% over the baseline level. During all surgical periods, a heating pad was used to maintain body temperature.

### Neurological scoring

Twenty-four hours after MCAO/R surgery, neurological score was graded as 0-4 based on Bederson’s method [25], with higher score reflecting more severe neurological defect. Grade 0: while being held suspended above the floor by the tail, mice extended both forelimbs toward the floor without other neurological deficits; grade 1: mice with consistent forelimb flexion to the injured hemisphere and no other abnormal behavior; grade 2: when being placed on a smooth surface, mice had consistently reduced resistance to lateral push on the shoulder toward the paretic side; grade 3: mice circled toward the paretic side; and grade 4: mice flaccid paralyses without spontaneous movements.

### 2, 3, 5-Triphenyltetrazolium chloride (TTC) staining

The whole brain was removed after mice were sacrificed at 24 h after reperfusion, and each brain was sliced into coronal sections (2 mm thickness). Brain slices were stained in TTC (Biosharp, Hefei, Anhui, China) at 37°C for 20 min, photographed with a digital camera, and analyzed using the ImageJ software (National Institutes of Health, Bethesda, MD, USA) [26]. Hemisphere volume was calculated as: ∑hemisphere area × slice thickness (2 mm). To avoid the influence of cerebral edema, infarction in each section was normalized to non-ischemic contralateral side and expressed as a percentage of the contralateral hemisphere using the formula: Infarct volume (%) = (contralateral volume - ipsilateral non-infarct volume)/contralateral volume × 100%.

### Brain edema determination

To estimate brain edema, brains were weighed prior to and 12 h after drying in a 110°C oven. Brain edema was calculated as percent of water content as following [27]: Brain water content (%) = (wet weight - dry weight)/wet weight × 100%.

### Evans blue extravasation

To evaluate the integrity of BBB [28], mice were injected with 3% Evans blue (2 mL/kg, Solarbio, Beijing, China) through femoral vein at 24 h after reperfusion. Mice were deeply anesthetized and perfused intracardially with phosphate-buffered saline (PBS) at 2 h after the Evans blue injection. The brain was photographed by a digital camera, and the ischemic hemispheres were separated after removing the cerebellum and brainstem. Each hemisphere was weighted and well homogenized in 3 mL formylamine. The homogenates were incubated at 56°C for 24 h, centrifuged at 3000 × g for 20 min, and Evans blue concentration in the supernatants was measured at 632 nm by a spectrophotometer. Data are presented as contents of Evans blue/hemisphere (μg/g).

### Cell culture and transfection

bEnd.3 cells were obtained from China Pharmaceutical University and cultured in high-glucose Dulbecco’s Modified Eagle’s Medium (DMEM, KeyGEM) supplied with 10% fetal bovine serum (Gibco, Grand Island, NY, USA), D-Glucose (4.5g/L), glutamine (2 mmol/L), penicillin (80 U/mL), streptomycin (0.08 mg/mL) and pyruvate (1 mmol/L) in humidified air (5% CO_2_) at 37°C. The cells at 70-80% confluence was transfected with mouse P-gp (GGATCCAGTCTAATAAGAA) siRNA (50 pmol per well (6-well), RiboBio, Guangzhou, Guangdong, China) or P-gp pcDNA3.1 (+) (Sangon Biotech, Shanghai, China) using the lipofectamine 2000 reagent (Invitrogen, Waltham, MA, USA) to silence or overexpress P-gp, respectively. The transfection efficiency was assayed 36 h after transfection by Western blot according to the manufacturer’s recommendation. The control cells were transfected with the same volume of NC siRNA (RiboBio) or NC pcDNA3.1 (+) (Sangon Biotech).

### OGD/R injury in endothelial cells

Confluent bEnd.3 cells were washed twice and subjected to OGD/R by replacing with serum-free low-glucose (D-Glucose, 1.0 g/L) DMEM in an anoxic incubator (95% N_2_ and 5% CO_2_) at 37°C for 2 h [29]. The cells were then transferred to a normoxic incubator (95% air, 5% CO_2_) and maintained in serum-free high-glucose DMEM for 24 h. In control group, cells were cultured in the chamber with serum-free high-glucose DMEM and normal culture conditions for the same period. The cells were treated with an autophagy inhibitor 3-MA (1 mmol/L) 1 h prior to OGD stimulation [30].

### Leukocyte adhesion and transendothelial migration

Leukocytes were isolated from bone marrow of mice and resuspended in high-glucose DMEM for experiments [31]. In leukocyte adhesion assay, leukocytes were labeled by 2′, 7′-bis-(2-carboxyethyl)-5-(and-6)-carboxyfluorescein (BCECF, MedChemExpress, 5 μg/mL) for 60 min. bEnd.3 cells were seeded on a 24-well plate and incubated with BCECF-labeled leukocytes for 30 min with a 10:1 ratio of leukocytes to endothelial cells [32]. Non-adherent leukocytes were removed by washing with culture media for 3 times. Adhesion cells were imaged by the fluorescence microscope (Ts2R, Nikon, Tokyo, Japan).

Leukocyte transendothelial cell migration assay was performed as previously described [33] with slight modification. bEnd.3 cells were seeded into the upper chamber of transwell inserts (5.0-μm pore size, 6.5-mm diameter, Corning Inc., Corning, NY, USA) at a density of 1×10^5^ cells/well and cultured in high-glucose DMEM medium with 10% fetal bovine serum. Culture medium (1.5 mL in the apical compartment and 2 mL in the basolateral compartment to avoid hydrostatic pressure) was replenished every 2 d. Approximately 6-7 d after seeded, bEnd.3 monolayers were obtained. Leukocyte (1×10^6^ cells in 0.2 mL of DMEM) were added to upper chamber and incubated for 4 h in bEnd.3 cells. Migrated leukocytes were counted from the lower chamber. Leukocyte transendothelial migration was quantitated as the number of leukocytes per square millimeter.

### Hematoxylin and eosin staining

Brains were harvested, fixed in 4% paraformaldehyde (PFA) for 24 h, dehydrated in graded ethanol, embedded in paraffin, and sectioned (6 μm). After deparaffinization and rehydration, sections were stained with Giles’ hematoxylin and eosin (H&E) and imaged on a fluorescent inverted microscope (BX53, Olympus, Tokyo, Japan).

### Immunohistochemistry staining

Paraffin sections (2 μm in thickness) were used for all staining. Briefly, following blocking endogenous peroxidase with PBS containing 3% hydrogen peroxide for 5 min, sections were stained with primary antibodies for neutrophils (myeloperoxidase, MPO, Cat#: bs-4943R, 1:200, Bioss, Boston, MA, USA), macrophages (F4/80, Cat#: 28463-1-AP, 1:1000, Proteintech, Wuhan, Hubei, China), intercellular adhesion molecules-1 (ICAM-1, Cat#: 10020-1-AP, 1:1000, Proteintech), vascular adhesion molecule-1 (VCAM-1, Cat#: BM4289, 1:1000, BOSTER, Wuhan, Hubei, China), and P-gp (Cat#: 13978S, 1:1000, Cell Signaling Technology, Danvers, MA, USA) at 4°C overnight. Then, sections were incubated with Immunohistochemical staining kit (DAKO, Carpinteria, CA, USA) for 1 h at room temperature. All stained sections were counterstained with Giles’ hematoxylin and imaged on a fluorescent inverted microscope (Ts2R, Nikon). To quantify P-gp expression levels, neutrophil and macrophages, randomly selected fields from the ischemic cortex were analyzed by using ImageJ software.

### Immunofluorescence staining

Mice were anesthetized 24 h after reperfusion and perfused intracardially with cold 4% PFA followed by perfusion with PBS. Brains were fixed with 4% PFA and embedded in optimal cutting temperature compound (OCT), and sectioned (12 μm) followed by blocking nonspecific staining with 8% goat serum for 2 h at room temperature. For culture cells, the cells were fixed in 4% PFA for 20 min and incubated with solution containing 0.3% Triton X-100 in PBS (PBST) containing 8% goat serum for 2 h at room temperature for non-specific staining blocking and permeabilization [34].

Tissue sections and cells were incubated with the primary antibodies against CD31 (endothelial cell, Cat#: ab28364, 1:200, Abcam, Boston, MA, USA) ICAM-1 (1:100), VCAM-1 (1:50), Claudin-5 (Cat#: AF5216, 1:100, Affinity Biosciences, Cincinnati, OH, USA), Occludin (Cat#: 27260-1-AP, 1:300, Proteintech), Zonula occludens-1 (ZO-1, Cat#: 21773-1-AP, 1:100, Proteintech), light chain 3 (LC3, Cat#: 14600-1-AP, 1:100, Proteintech) and glucocorticoid receptor (GR, Cat#: 24050-1-AP, 1:100, Proteintech) at 4°C overnight. Following 3 washes with PBST, sections or cells were incubated with Cy3-conjugated goat anti-mouse IgG (Cat#: bs-0296G-Cy3, 1:200, Bioss) and FITC-conjugated goat anti-rabbit IgG (Cat#: bs-0295G-FITC, 1:200, Bioss) for 2 h at room temperature, washed with PBST 3 times and counterstained with 4, 6-diamidino-2-phenylindole (DAPI, Beyotime Institute of Bio-technology, Shanghai, China) for 30 min at room temperature. Stained images were captured by a laser confocal microscope (FV3000, Olympus) or a fluorescent microscope (BX53, Olympus), and analyzed using ImageJ software.

### Real time quantitative reverse transcription polymerase chain reaction (RT-PCR) assay

Total RNA was extracted using the RNA isolater (Vazyme Biotech, Nanjing, Jiangsu, China) and was transcribed into cDNA using a HiScript II Q RT SuperMix (Vazyme Biotech). Real time PCR was performed using quantitative PCR (Mastercycler ep realplex, Eppendorf, Germany) with a fluorescent dye (SYBR Green I). mRNA levels were normalized to β-actin of the same samples and were reported as fold changes relative to sham or control treatment [35]. The primers (Sangon Biotech) were provided in the Supplementary Table 1.

**Figure 1.**
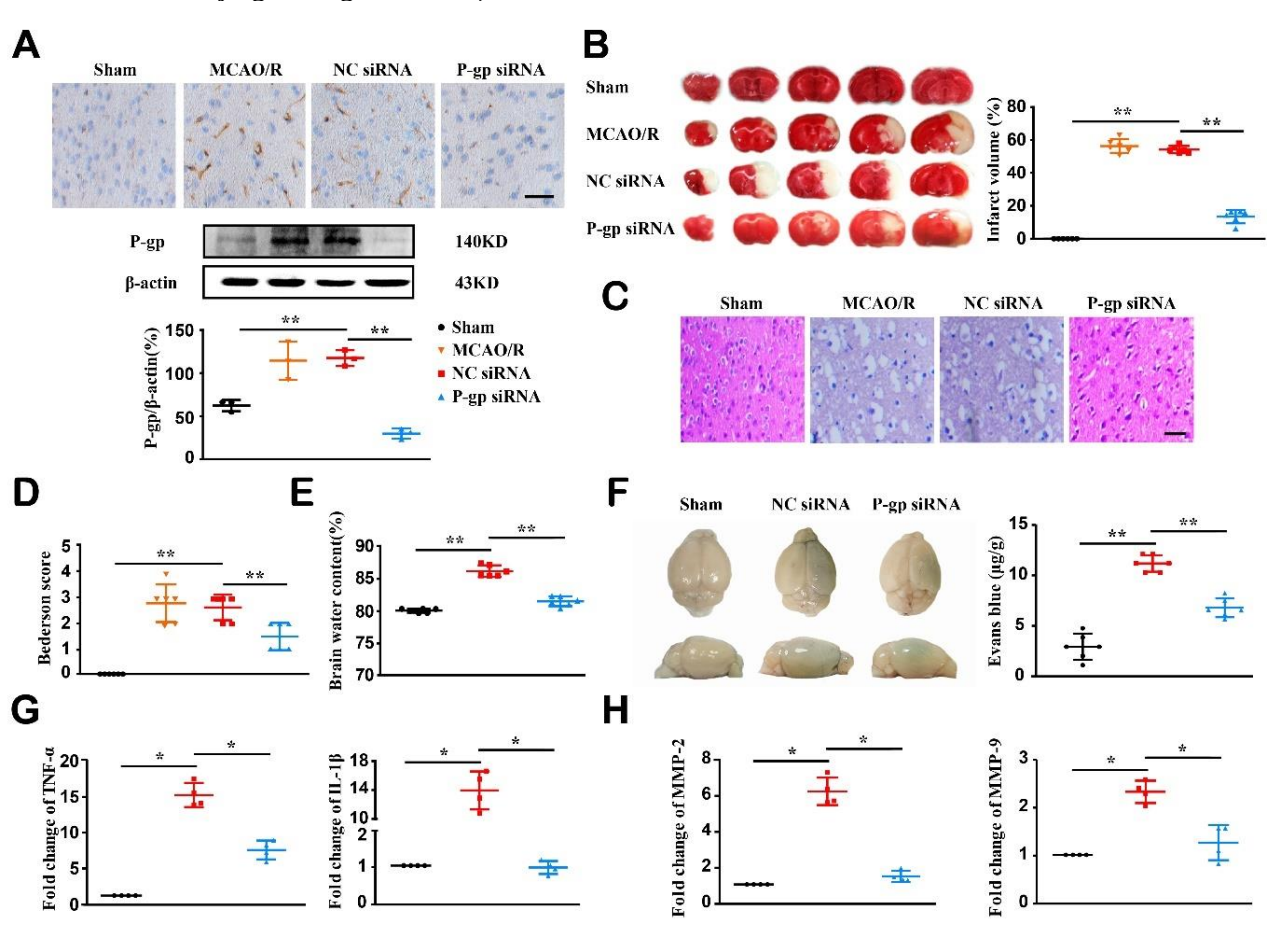
**P-glycoprotein silence reduces ischemic infarction and brain inflammation in experimental ischemic stroke**. Mice were intracerebroventricularly injected with P-glycoprotein (P-gp) or negative control (NC) siRNA (1.5 μL/10 g body weight) 48 h prior to MCAO/R surgery. Twenty-four hours after the surgery, mice were subjected to neurological behavior testing, and brains were harvested for histological analyses, real-time PCR gene expression and Western-blotting analyses. (**A**) Immunohistochemical localization (top) and Western-blotting quantification (middle and bottom) of P-gp levels in brain cortex (n = 3). (**B**) Representative TTC staining images and quantification of infarct volume (n = 6). (**C**) Representative H&E images for brain vacuolization. (**D**) Neurological behavior assessed by Bederson score (n = 6). (E, F) Blood-brain barrier permeability assessed by Brain water content (E) and Evans blue extravasation (F) (n = 6). (G, H) mRNA expression levels of TNF-α, IL-1β, MMP-2, and MMP-9 measured via quantitative real-time PCR assay as fold changes relative to sham treatment (n = 4). Scale bars, 40 μm. One-way ANOVA followed by the post hoc Tukey test for A, E, and F. Mann-Whitney test for B, D, G, and H. All data are mean ± SD, ^*^P<0.05, ^**^P<0.01 between two groups.

### Western-blotting analysis

Total protein was extracted using RIPA lysis containing protease and phosphatase inhibitor cocktail (APEXBIO, Houston, TE, USA). Protein concentrations were determined by BCA assay. Proteins were separated by SDS-PAGE and transferred onto polyvinylidene fluoride (PVDF) membranes. After blocking with 5% non-fat milk in Tris-buffered saline with 0.05% Tween 20 (TBST) for 1.5 h at room temperature, the membrane was incubated with antibodies against P-gp (1:500), Claudin-5 (1:500), Ocluddin (1:1000), ZO-1 (1:1000), LC3 (1:1000), mTOR (Cat#: 66888-1-Ig, 1:1000, Proteintech), p-mTOR (Cat#: ab134903, 1:10000, Abcam), Akt (Cat#: ab74117, 1:10000, Abcam), p-Akt (Cat#: AP1208, 1:500, ABclonal, Wuhan, Hubei, China) and β-actin (Cat#: AC026, 1:8000, ABclonal) at 4°C overnight. After 3 times washes with TBST, the blots were incubated with HRP goat anti-rabbit IgG (H + L) (Cat#: AS014, 1:8000, ABclonal) or HRP-labeled goat anti-mouse IgG (H + L) (Cat#: AS003, 1:8000, ABclonal) for 2 h at room temperature. The bands were visualized using the enhanced chemiluminescence (ECL) reagents (Affinity Biosciences) and were quantified with ImageJ software. The protein expression levels were expressed as the percentage of β-actin.

### Brain dexamethasone concentration determination

To evaluate the function of P-gp on glucocorticoid transport, mice were injected with exogenous glucocorticoid (dexamethasone sodium phosphate, 4 mg/kg, Yuanye Biotech, Shanghai, China) through femoral vein at 24 h after MCAO/R. Brains were harvested and ischemic hemisphere was separated at 5 min after the injection. Brain was homogenized with pure water with a mass/volume ratio of 1:3. Dexamethasone concentration in homogenates was determined by a UPLC-MS/MS system (Waters UPLS-AB Qtrap 6500 plus, Framingham, MA, USA) and given as ng/g ischemic brain tissue.

### Statistical analysis

All statistical analyses were performed using IBM SPSS Statistics 19.0 software. The distribution of all data was tested for normality via a One-Sample Kolmogorov-Smirnov test. Statistical differences among the groups were analyzed by One-Way Analysis of Variance (ANOVA) with post hoc Tukey tests if data were normally distributed and consistent with homogeneity of variance among the experimental groups. The Mann-Whitney test was used to the compare ranks if the data were not normally distributed or not consistent with homogeneity of variance. The difference was considered significant at *P*<0.05. All data are presented as the means ± SD.

**Figure 2.**
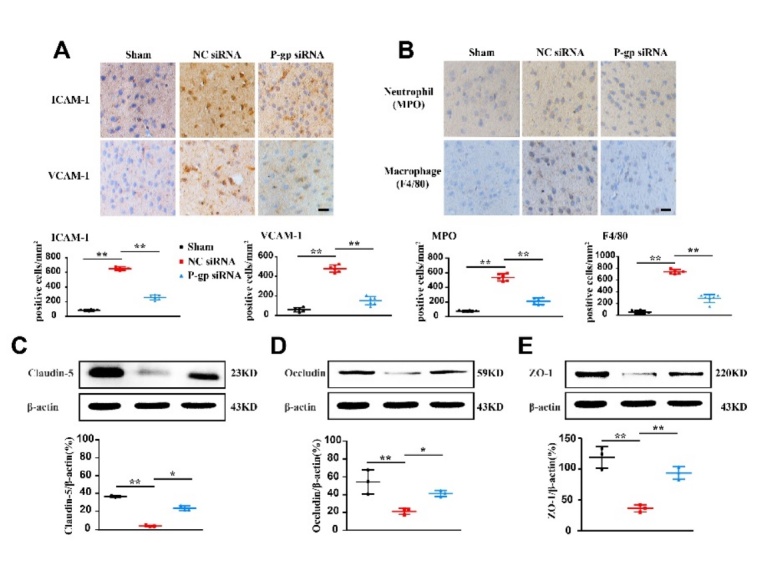
**P-glycoprotein silence attenuates adhesion molecule expression, myeloid cell accumulation and blood-brain barrier damage in experimental ischemic stroke**. Mice were intracerebroventricularly injected with P-glycoprotein (P-gp) or negative control (NC) siRNA (1.5 μL/10 g body weight) 48 h prior to MCAO/R surgery. Twenty-four hours after the surgery, brains were harvested for immunohistochemical and Western-blotting assays. (**A**) Immunohistochemical localization and quantification of ICAM-1 and VCAM-1 expression (n = 6). (**B**) Representative immunostaining images and quantification of neutrophiles (MPO) and macrophages (F4/80) (n = 6). (C-E) Representative Western-blotting images and quantification of tight junction proteins (Claudin-5, Occludin, and ZO-1) (n = 3). Scale bars: 40 μm. One-way ANOVA followed by the post hoc Tukey test. All data are mean ± SD, ^*^P<0.05, ^**^P<0.01 between two groups.

**Figure 3.**
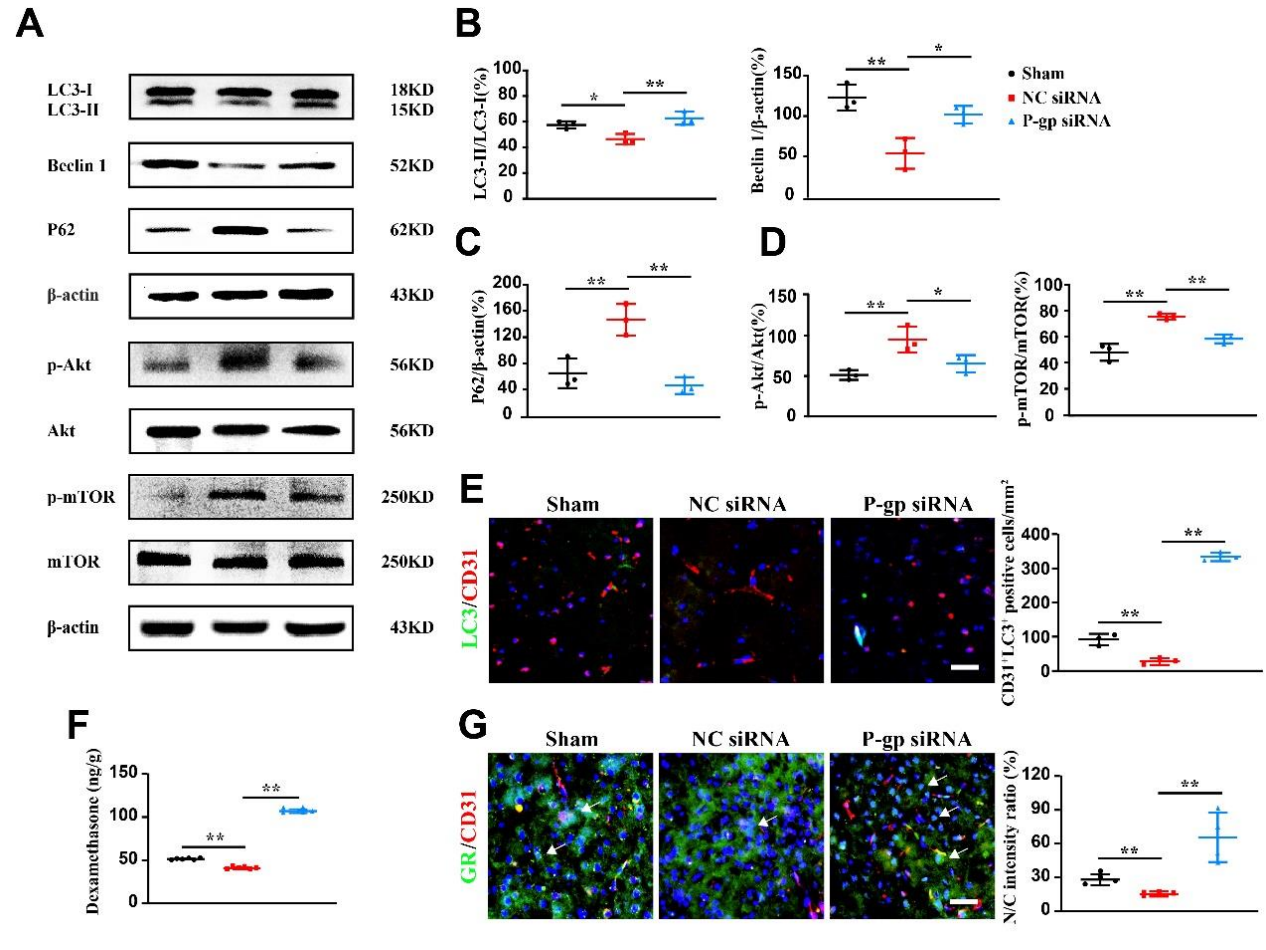
**P-glycoprotein silence activates endothelial autophagy in experimental ischemic stroke by reducing Akt/mTOR signal activity**. Mice were intracerebroventricularly injected with P-glycoprotein (P-gp) or negative control (NC) siRNA (1.5 μL/10 g body weight) 48 h prior to MCAO/R surgery. Twenty-four hours after the surgery, brains were harvested for immunofluorescence, dexamethasone determination and Western-blotting assays. (**A**) Representative immunoblots of autophagic proteins and Akt/mTOR. (B-D) Quantification of the ratio of LC3-II to LC3-1 (LC3-II/LC3-1, B), Beclin 1 (B) and P62 (C), the ratios of phosphorylated Akt (p-Akt) to total Akt (p-Akt/Akt, D) and phosphorylated mTOR (p-mTOR) to total mTOR (p-mTOR/mTOR, D) in the cortex of mouse brains (n = 3). (**E**) Immunofluorescence colocalization and quantification of autophagic protein LC3 in the brain blood vessels (CD31) (n = 3). (**F**) Quantification of dexamethasone levels (n = 6). (**G**) Representative immunofluorescence images and quantification of nuclear and cytoplasmic localization (ratio of nuclear to cytoplasmic location) of Glucocorticoid receptor (GR) (n = 4). Scale bars, 40 μm. One-way ANOVA followed by the post hoc Tukey test for B, C, and D. Mann-Whitney test for E and G. All data are mean ± SD, ^*^P<0.05, ^**^P<0.01 between two groups.

## RESULTS

### P-gp silence alleviates ischemic injury and inhibits inflammatory response

Mice underwent the MCAO surgery were included for successful occlusion. P-gp expression was substantially increased at ischemic hemisphere 24 h following MCAO/R surgery. Treatment with P-gp siRNA significantly reduced P-gp expression in ischemic brain as compared to NC siRNA treatment (Fig. 1A, *P*<0.01). Treatment with NC siRNA did not impact P-gp expression, infarct volume, and brain histopathology. Therefore, NC siRNA + MCAO/R mice were used to observe P-gp function in following experiments as model control. P-gp siRNA pretreatment reduced infarct volume, attenuated neurological impairment, and improved histopathological damage as compared to NC siRNA treatment after MCAO/R (Fig. 1B-1D, *P*<0.01).

Ischemic stroke breaks down BBB, leading to the entry of serum proteins into brain fluid and edema formation [36]. Twenty-four hours after the reperfusion, P-gp siRNA treatment dramatically mitigated brain edema and Evans blue extravasation in the ischemic area from mice as compared to NC siRNA treatment (Fig. 1E, 1F; *P*<0.01).

MMP-2 and MMP-9 degrade collagen IV that is a major component of basal lamina, and ultimately disrupt BBB [37]. In RT-PCR assays, mRNA levels of TNF-α, IL-1β, MMP-2, and MMP-9 were remarkably elevated following ischemic stroke. However, these alterations were blunted by P-gp siRNA pretreatment (Fig. 1G-1H, *P*<0.05).

**Figure 4.**
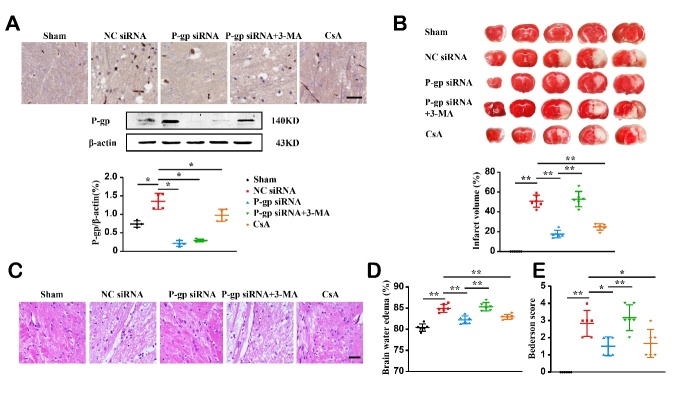
**Pharmacologically autophagy inhibitor counteracts the influence of P-glycoprotein silence on, and pharmacological P-glycoprotein inhibitor attenuates experimental ischemic stroke**. Inhibitors to autophagy (3-MA, intracerebroventricular, 15 μg/mouse) and P-glycoprotein (CsA, intravenous, 22 mg/kg body weight) were given to mice 30 min prior to and immediately the MCAO/R surgery, respectively. Twenty-four hours after the surgery, mice were sacrificed, and brains were collected for histological and Western-blotting analyses. (**A**) Immunostaining (top) and Western-blotting quantification (middle and bottom) of P-glycoprotein expression (n = 4). (**B**) Representative images of TTC-stained brain sections and quantification of infarct volume (n = 6). (**C**) Representative images for brain vacuolization revealed by H&E staining. (**D**) Blood-brain barrier permeability assessed by brain water content (n = 6). (**E**) Neurological defects assessed by Bederson score (n = 6). Scale bars, 40 μm. One-way ANOVA followed by the post hoc Tukey test for B, C, and D. Mann-Whitney test for E, F, and G. All data are mean ± SD, ^*^P<0.05, ^**^P<0.01 between two groups.

### P-gp silence inhibits BBB adhesion molecule expression and leukocyte accumulation, and maintains tight junction

ICAM-1 and VCAM-1 are important for recruiting leukocytes into inflamed brain. ICAM-1 and VCAM-1 levels were significantly lower in the ischemic area of mice treated with P-gp siRNA than those in mice treated with NC siRNA (Fig. 2A, *P*<0.01). In tissue immunostaining, neutrophils (MPO) and macrophages (F4/80) were observed in the infarct site 24 h after MCAO/R. However, P-gp siRNA treatment substantially reduced the densities of both neutrophils and macrophages as compared to NC siRNA treatment (Fig. 2B, *P*<0.01).

In the CNS, tight junctions form a barrier to limit paracellular permeability. In Western-blotting analysis, the expression levels of TJPs including Occludin, Claudin-5, and ZO-1 were largely reduced in ischemic brain of NC siRNA-treated MCAO/R mice. In contrast, P-gp siRNA treatment restored the expression levels of all proteins in ischemic area (Fig. 2C-2E, *P*<0.05, *P*<0.01). These results indicate that silencing P-gp reduced BBB permeability by increasing TJPs expression.

### P-gp silence activates endothelial autophagy via Akt/mTOR signal pathway

To determine the influence of P-gp on endothelial autophagy, we assessed the expression of microtubule-associated protein LC3 and autophagy adapter protein P62, two well established markers for autophagy activation [38, 39]. In Western-blotting analysis, the ratio of LC3-II/LC3-I and the expression of Beclin 1 were dramatically increased in P-gp siRNA-treated mice, with reduced expression of P62, as compared to NC siRNA treatment (Fig. 3A-3C, *P*<0.05, *P*<0.01). In immunofluorescence staining, P-gp knockdown also dramatically enhanced LC3 expression in endothelial cells (CD31^+^) (Fig. 3E, *P*<0.01).

Akt/mTOR activity suppressed autophagy [40]. As indicated by increased ratio of phosphorylated to total Akt and mTOR, Akt/mTOR activity was elevated in ischemic brain. P-gp siRNA treatment markedly reduced the phosphorylated levels of Akt and mTOR in ischemic brain, suggesting the suppression of Akt/mTOR by P-gp silence (Fig. 3D, *P*<0.01). Glucocorticoid binds to glucocorticoid receptor (GR), induces GR nuclear translocation and thus inhibits Akt/mTOR activity [41]. In ischemic brain from NC siRNA-treated mice, exogenous glucocorticoid (dexamethasone) levels and GR nuclear translocation were significantly reduced as compared to sham mice. In contrast, P-gp siRNA increased dexamethasone levels and GR nuclear translocation in ischemic brain (Fig. 3F, 3G; *P*<0.01).

**Figure 5.**
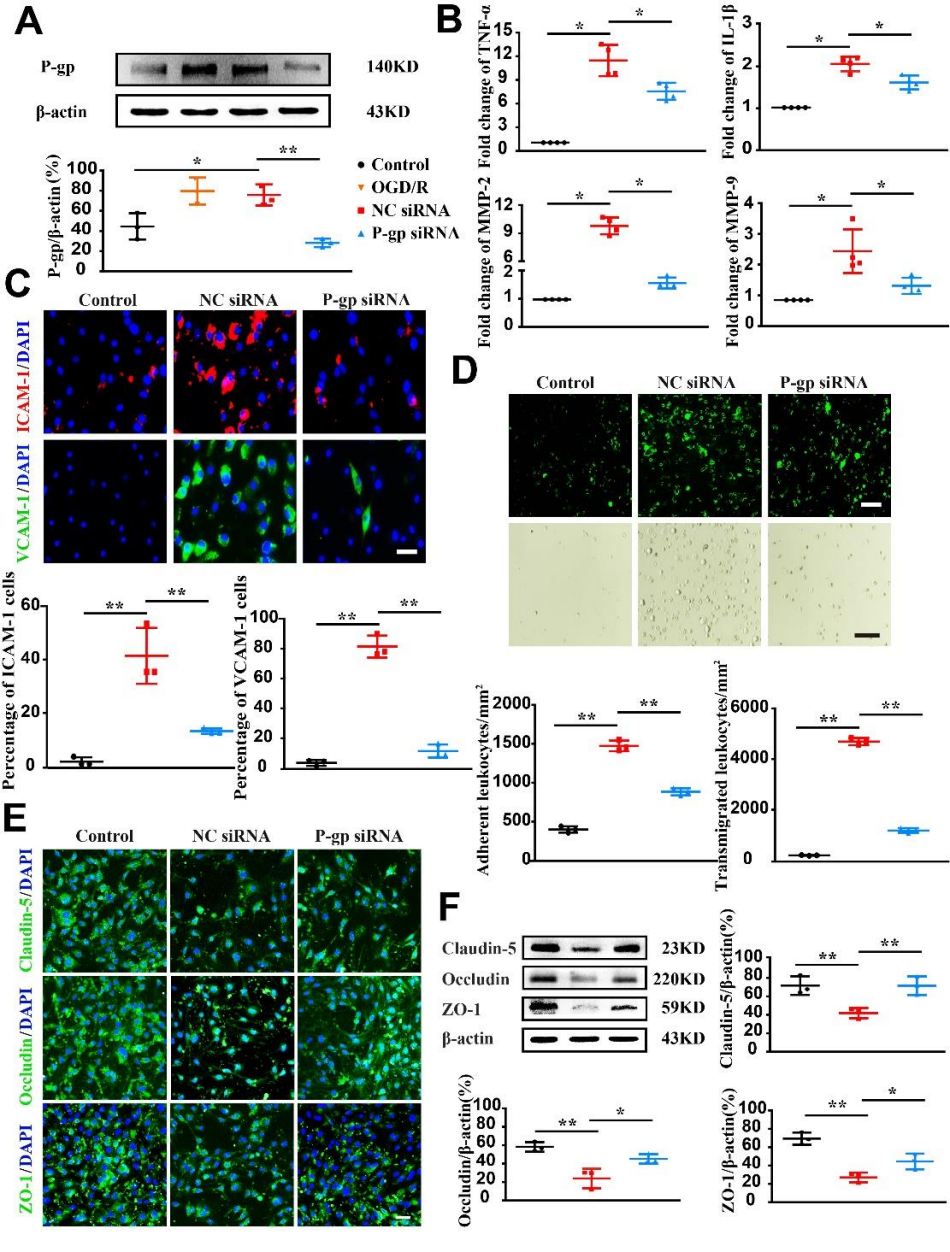
**P-glycoprotein silence alleviates endothelial dysfunction following oxygen glucose deprivation/reoxygenation**. Endothelial cells (bEnd.3) were transfected with P-glycoprotein (P-gp) or negative control (NC) siRNA, or un-transfected, and then subjected to either oxygen glucose deprivation/reoxygenation (OGD/R) treatment or normal culture conditions. Twenty-four hours thereafter, cells were harvested for immunofluorescence staining, adhesion and transendothelial migration, real-time PCR gene expression and Western-blotting analyses. (**A**) Representative Western-blotting images and quantification of P-gp levels (n = 3). (**B**) Quantification of mRNA levels for TNF-α, IL-1β, MMP-2, and MMP-9 via quantitative real-time PCR assay (n = 4). (**C**) Representative immunofluorescence staining images and quantification of ICAM-1 and VCAM-1 expression (n = 3). (**D**) Representative images and quantification for fluorochrome-labeled leukocyte adhesion to endothelial cells and transendothelial migration (n = 3). (**E**) Representative immunofluorescence staining images for tight junction proteins (Claudin-5, Occludin, and ZO-1). (**F**) Representative immunoblots and quantification for the expressions of Claudin-5, Occludin, and ZO-1 (n = 3). Scale bars, 40 μm. One-way ANOVA followed by the post hoc Tukey test for A, E, and F. Mann-Whitney test for B, C, and D. All data are mean ± SD, ^*^P<0.05, ^**^P<0.01 between two groups.

**Figure 6.**
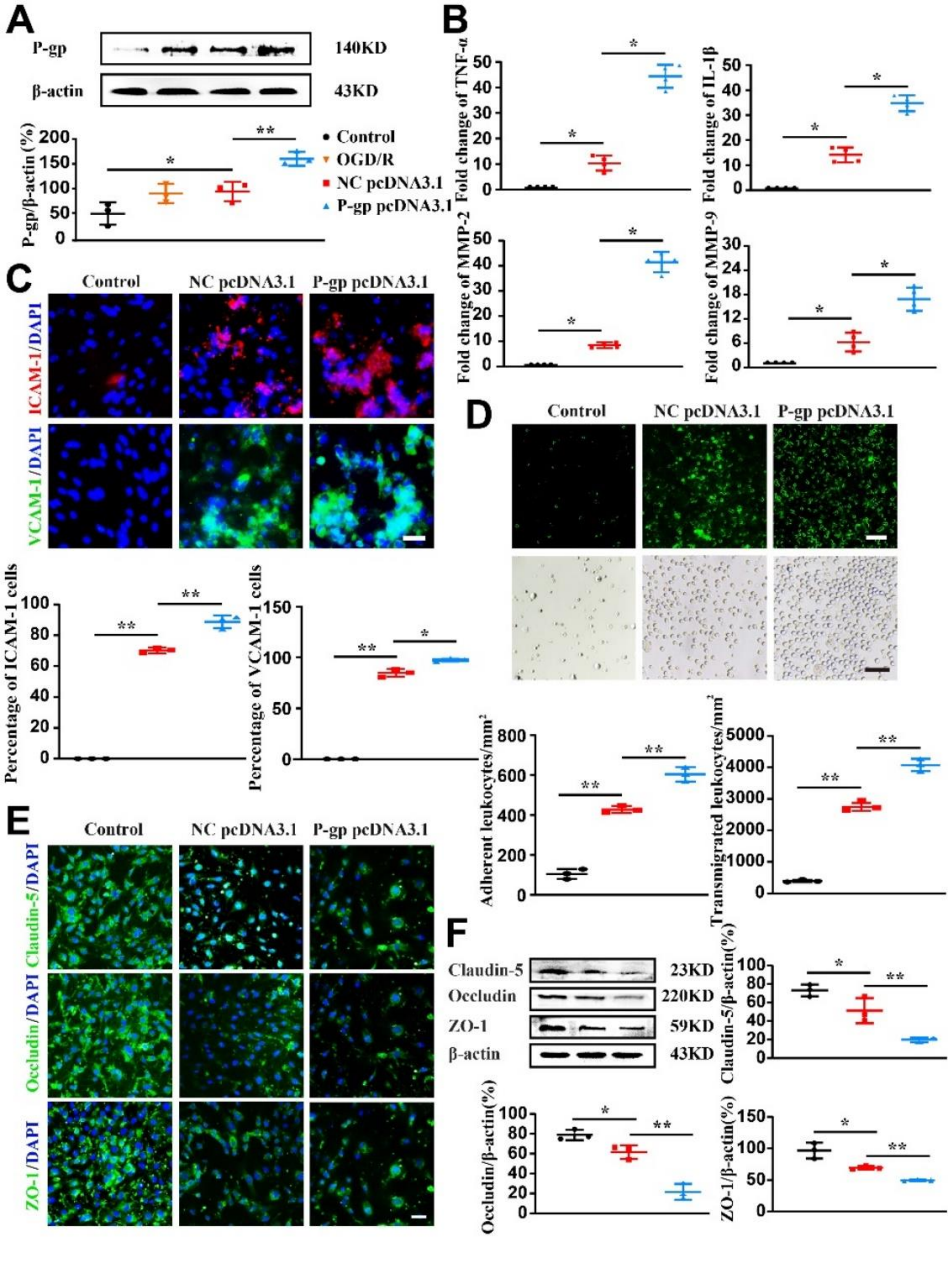
**P-glycoprotein overexpression exacerbates endothelial dysfunction following oxygen glucose deprivation/reoxygenation**. Endothelial cells (bEnd.3) were transfected with P-glycoprotein (P-gp) pcDNA3.1 plasmid, negative control (NC) pcDNA3.1 plasmid or un-transfected, and then subjected to either oxygen glucose deprivation/reoxygenation (OGD/R) treatment or normal culture conditions. Twenty-four hours thereafter, cells were harvested for immunofluorescence staining, adhesion and transendothelial migration, real-time PCR gene expression and Western-blotting analyses. (**A**) Representative Western-blotting images and quantification of P-gp protein levels (n = 3). (**B**) Quantification of mRNA levels for TNF-α, IL-1β, MMP-2, and MMP-9 (n = 4). (**C**) Representative immunofluorescence staining images and quantification of ICAM-1 and VCAM-1-expression (n=3). (**D**) Images and quantification of fluorochrome-labeled leukocyte adhesion to endothelial cells and transendothelial migration (n = 3). (**E**) Representative immunofluorescence staining images for tight junction proteins (Claudin-5, Occludin, and ZO-1). (**F**) Representative Western-blotting images and quantification of the expressions of Claudin-5, Occludin, and ZO-1 (n = 3). Scale bars, 40 μm. One-way ANOVA followed by the post hoc Tukey test for A, D, E, and F. Mann-Whitney test for B and C. All data are shown as mean ± SD, ^*^P<0.05, ^**^P<0.01 between two groups.

**Figure 7.**
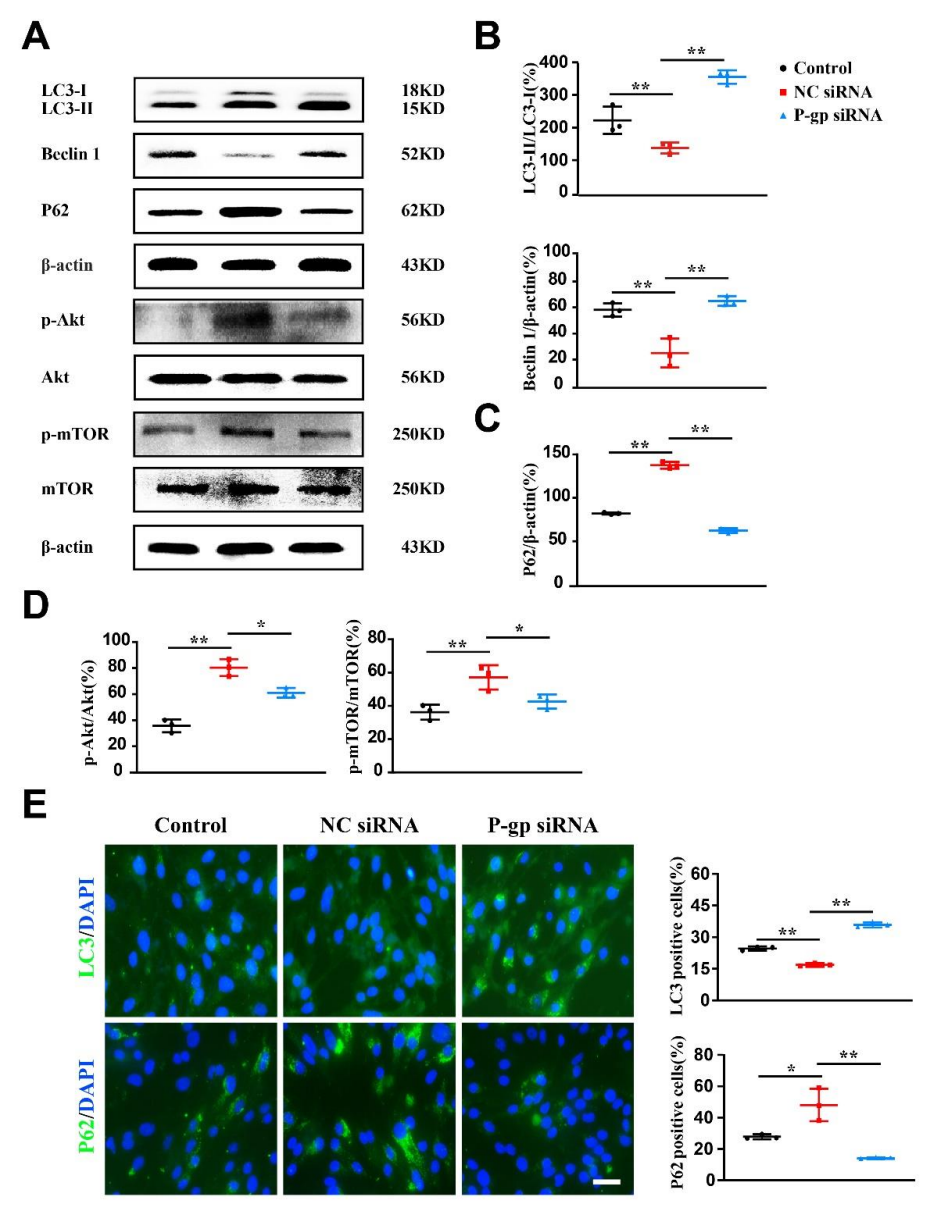
**P-glycoprotein silence activates autophagy and inhibits Akt/mTOR activity in endothelial cells following oxygen glucose deprivation/reoxygenation**. Endothelial cells (bEnd.3) were transfected with P-glycoprotein (P-gp) or negative (NC) siRNA or un-transfected, and then subjected to either oxygen glucose deprivation/reoxygenation (OGD/R) treatment or normal culture conditions. Twenty-four hours thereafter, cells were harvested for immunofluorescence staining and Western-blotting analyses. (**A**) Representative Western-blotting images for autophagic proteins, total and phosphorylated Akt and mTOR proteins. (B-D) Quantification of the ratio of LC3-II to LC3-1 (LC3-II/LC3-1, B), Beclin 1 (B) and P62 (C), the ratios of phosphorylated Akt (p-Akt) to total Akt (p-Akt/Akt, D) and phosphorylated mTOR (p-mTOR) to total mTOR (p-mTOR/mTOR, D) (n = 3). (**E**) Representative images and quantification of LC3 and P62 immunofluorescence staining (n = 3). Scale bars, 40 μm. One-way ANOVA followed by the post hoc Tukey test for B, D, and E. Mann-Whitney test for C. All data are shown as mean ± SD, ^*^P<0.05, ^**^P<0.01 between two groups.

### Pharmacologically inhibiting P-gp attenuates, while autophagy inhibitor negates the effects of P-gp silence on experimental stroke outcomes

To determine whether autophagy contributes to the suppression of ischemic stroke by P-gp inhibition, an autophagy inhibitor 3-MA was given to mice 30 min prior to stoke induction. 3-MA treatment did not alter P-gp expression in ischemic brain of P-gp siRNA-treated mice. However, inhibiting autophagy by 3-MA counteracted the effects of P-gp siRNA treatment on ischemic stroke outcome as indicated by enlarged infraction size, increased brain edema, worsened neurological defects and brain histopathology (Fig. 4B-4E, *P*<0.05, *P*<0.01).

Additionally, similar to P-gp siRNA silence, pharmacological inhibition of P-gp with CsA significantly attenuated P-gp expression levels in ischemic brain as compared to vehicle treatment (Fig. 4A, *P*<0.05). Histologically, CsA treatment reduced infarct volume, brain edema, neurological behavior defects and brain histopathology induced by MCAO/R (Fig. 4B-4E, *P*<0.05, *P*<0.01).

### P-gp silence alleviates endothelial dysfunction

Under OGD/R condition, P-gp expression was markedly elevated in endothelial cells (bEnd.3 cells). P-gp knockdown dramatically reduced P-gp expression as compared to NC siRNA incubation (Fig. 5A, *P*<0.01). Preincubation with P-gp siRNA inhibited mRNA expressions of TNF-α, IL-1β, MMP-2, and MMP-9 in endothelial cells induced by OGD/R (Fig. 5B, *P*<0.05). Consistent with *in vivo* findings, P-gp knockdown substantially downregulated the expression of ICAM-1 and VCAM-1 on endothelial cells following OGD/R treatment (Fig. 5C, *P*<0.01). P-gp silence also mitigated leukocyte adhesion and transendothelial migration (Fig. 5D, *P*<0.01), while increased the expression levels of TJPs (Claudin-5, Occludin and ZO-1) in endothelial cells undergoing OGD/R (Fig. 5E, 5F and Supplementary Fig. 3; *P*<0.05, *P*<0.01).

### P-gp overexpression exacerbates endothelial dysfunction

To examine the effect of P-gp overexpression, endothelial cells were transfected with P-gp pcDNA3.1 plasmid to forcefully express P-gp or with pcDNA3.1 plasmid serving the NC. P-gp pcDNA3.1 transfection remarkably upregulated P-gp expression (Fig. 6A, *P*<0.01) as compared to NC plasmid pcDNA3.1. P-gp overexpression increased mRNA expression levels of TNF-α, IL-1β, MMP-2, and MMP-9 following OGD/R treatment as compared to NC plasmid pcDNA3.1 (Fig. 6B; *P*<0.05). Additionally, P-gp overexpression also increased adhesion molecules expression, augmented leukocytes adhesion and transmigration (Fig. 6C, 6D; *P*<0.05, *P*<0.01), whereas reduced the expression levels of Claudin-5, Occludin, and ZO-1 in endothelial cells following OGD/R (Fig. 6E, 6F and Supplementary Fig. 3; *P*<0.05, *P*<0.01).

### P-gp silence activates autophagy in endothelial cells by inhibiting Akt/mTOR pathway activity

To assess the influence of P-gp on autophagy and potential mechanisms, endothelial cells were transfected with P-gp or NC siRNA followed by OGD/R treatment. In Western-blotting and immunofluorescence staining analyses, P-gp silence dramatically increased the the ratio of LC3-II/LC3-I as well as Beclin 1 levels, whereas reduced P62 levels (Fig. 7A-7C, 7E; *P*<0.01). These data indicate that P-gp silence led to autophagy activation to protect endothelial cells against OGD/R-induced cellular injury. Further, P-gp silence largely diminished the phosphorylation of Akt and mTOR protein signaling (Fig. 7D, *P*<0.05), suggesting that P-gp silence may activate autophagy by inhibiting Akt/mTOR signaling activity.

### Pharmacological inhibition of autophagy abrogates the effects of P-gp silence on endothelial tight junction protein expression

**Figure 8.**
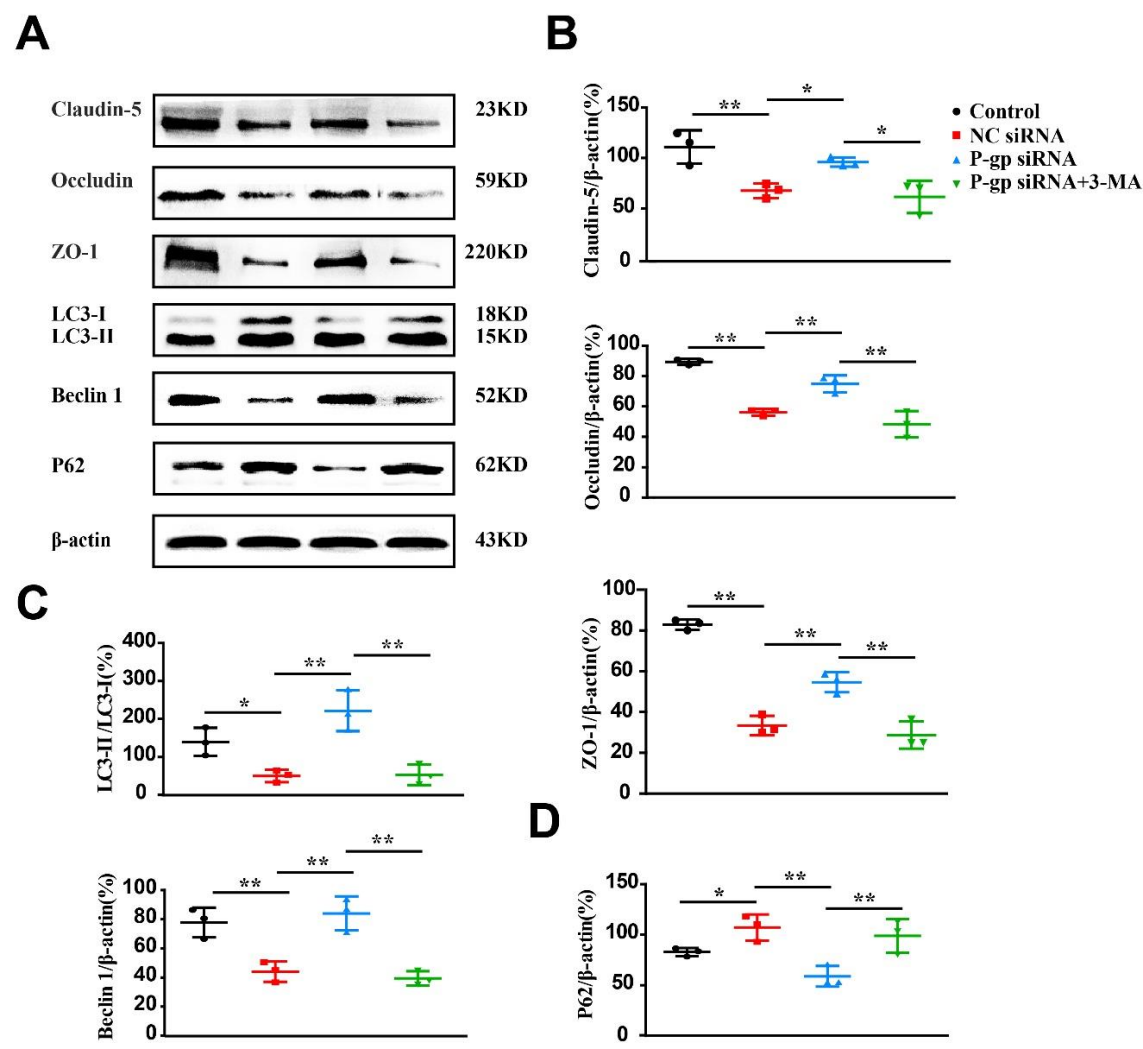
**Pharmacological autophagy inhibitor counteracts the augmentation of tight junction proteins by P-glycoprotein silence in endothelial cells following oxygen glucose deprivation/reoxygenation**. Endothelial cells (bEnd.3) were transfected with P-gp or negative (NC) siRNA, P-glycoprotein (P-gp) siRNA plus 3-methyladenine (3-MA), or un-transfected, and then subjected to either oxygen glucose deprivation/reoxygenation (OGD/R) treatment or normal culture conditions. Twenty-four hours thereafter, cells were harvested for Western-blotting analysis. (**A**) Representative immunoblot images of tight junction proteins and autophagic proteins. (B-D) Quantification of tight junction proteins (Claudin-5, Occludin, and ZO-1, B), ratio of LC3-II to LC3-I (LC3-II/LC3-1, C), Beclin 1 (C) and P62 (D) (n = 3). One-way ANOVA followed by the post hoc Tukey test. All data are shown as mean ± SD, ^*^P<0.05, ^**^P<0.01 between two groups.

Although OGD/R treatment dramatically reduced TJPs levels in endothelial cells, P-gp silence increased the suppression of TJPs expression following OGD/R as compared to NC siRNA treatment (Fig. 8A-8B, *P*<0.05, *P*<0.01). When endothelial cells were incubated with a small molecule inhibitor 3-MA, the upregulation of TJPs by P-gp silence was almost ablated (Fig. 8A-8B, *P*<0.05, *P*<0.01). Expectedly, 3-MA treatment reduced the ratio of LC3-II/LC3-I and Beclin 1 expression (Fig. 8C, *P*<0.01), whereas increased P62 expression (Fig. 8D, *P*<0.01). These results indicate that autophagy activation by P-gp silence may protect OGD/R-induced endothelial dysfunction.

## DISCUSSION

Despite advances in our understanding of P-gp in multiple drug resistance, the role and underlying mechanisms in BBB dysfunction induced by ischemic stroke remain largely unknown. In this study, we found increased expression of P-gp in experimental ischemic stroke both *in vivo* and *in vitro*. P-gp silence or pharmacological inhibition alleviated ischemic stroke by improving the integrity and function of BBB. Therefore, P-gp overexpression caused BBB dysfunction and exacerbated stroke outcome by destroying TJPs and worsening inflammatory response.

P-gp is involved in the pathogenesis of certain CNS diseases by regulating inflammatory cytokine expression and immune cell infiltration [11, 12]. Though brain infarct volume was reduced in P-gp-deficient mice following MCAO/R [42], the influence of P-gp on BBB has not been comprehensively studied. BBB is formed by BMVECs, pericytes and astrocytes, and disrupted by ischemic stroke, leading to increased permeability thereby further aggravating cerebral ischemic injury [43, 44]. In consistent with previous study, we found that P-gp expression was remarkably elevated after ischemic stroke both in ischemic brain and in OGD/R-injured endothelial cells. To study the role of P-gp in ischemic stroke, we silenced P-gp by siRNA via i.c.v injection in mice or incubation with endothelial cells *in vitro*, or overexpressed P-gp by transfecting endothelial cells with P-gp pcDNA3.1. We showed that P-gp siRNA relived acute stroke injury, including reduction in infarct volume, improvement in neurological behaviors, and decrease in brain edema. Similar effects were also observed using a P-gp inhibitor CsA. Therefore, elevated P-gp expression induced by ischemic stroke is vital for aggravating acute stoke injury.

P-gp is mainly expressed on BMVECs of BBB that functions as a semipermeable barrier between the CNS and the peripheral circulation [45, 46]. In immortalized mouse brain endothelial cell line bEnd.3 cells, P-gp expression was obviously increased following OGD/R. BMVECs are rapidly activated after stroke, secrete proinflammatory cytokines [47, 48], express high level of adhesion molecules, produce extracellular matrix-degrading MMPs, disturb basement membranes, and weaken blood vessels [49]. Although all these factors further induce leukocyte migration and exacerbate ischemic stroke [50-52], whether P-gp is involved in this process still remains unclear. Compared with NC siRNA treatment, P-gp silence diminished mRNA expressions of TNF-α, IL-1β, MMP-2, and MMP-9, with expressions of ICAM-1 and VCAM-1 in mice after MCAO/R and endothelial cells after OGD/R injury. Leukocyte adhesion and migration were also inhibited by P-gp silence. Conversely, P-gp overexpression deteriorated expressions of proinflammatory cytokines, MMPs, and adhesion molecules in endothelial cells after OGD/R injury. Therefore, P-gp may contribute to BBB damage in ischemic stroke by modulating leukocyte adhesion on and transmigration through BMVECs.

TJPs including ZO-1, Claudin-5, and Occludin are important in regulating the integrity and permeability of BBB and are disrupted and redistributed after ischemic stroke [53, 54]. We showed that P-gp silence decreased BBB permeability as evidenced by reduced Evans blue extravasation into brain. P-gp silence upregulated whereas P-gp overexpression downregulated TJPs expression levels.

Autophagy is a crucial degradation pathway for maintaining cellular and energy homoeostasis and protecting cell death [55]. Autophagy inducers such as rapamycin and lithium carbonate have been shown to maintain BBB integrity in rats exposed to epilepsy and traumatic brain injury [17, 56, 57]. Autophagy activation also prevented BMVEC from apoptosis after OGD/R and maintain BBB integrity in ischemic stroke [19, 58]. P-gp silence activated endothelial autophagy as indicated by increased LC3-II/LC3-I ratio and Beclin 1 levels as well as decreased P62 levels. Further, P-gp silence inhibited the activity of Akt/mTOR pathway, which is important for endothelial autophagy. Conversely, autophagy inhibitor 3-MA abolished effects of P-gp silence on acute ischemic stroke injury and TJPs expression. The principal mechanism may be associated with transport function of P-gp. In ischemic brain, P-gp limits the brain delivery of glucocorticoid, leading to GR nuclear translocation inhibition, which results in Akt/mTOR signaling activation. We found that P-gp silence increased exogenous glucocorticoid concentration in ischemic brain and promoted GR nuclear translocation.

In conclusion, we demonstrated that elevated P-gp levels following ischemic stroke exacerbated BBB breakdown and brain inflammatory response by increasing Akt/mTOR activity and suppressing autophagy activation. Our findings help understand the role and underlying mechanisms of P-gp in brain inflammatory response and BBB integrity following ischemic stroke.

## Supplementary Materials

The Supplementary data can be found online at: www.aginganddisease.org/EN/10.14336/AD.2022.0225.


