# Erratum to “P-Glycoprotein Aggravates Blood Brain Barrier Dysfunction in Experimental Ischemic Stroke by Inhibiting Endothelial Autophagy”

**DOI:** 10.14336/AD.2025.10326

**Published:** 2025-03-26

**Authors:** Liangliang Huang, Yan Chen, Rui Liu, Binbin Li, Xuan Fei, Xiang Li, Ge Liu, Yunman Li, Baohui Xu, Weirong Fang

In our article “P-Glycoprotein Aggravates Blood Brain Barrier Dysfunction in Experimental Ischemic Stroke by Inhibiting Endothelial Autophagy” published in the October 2022 issue of Aging and Disease [Aging Dis. 2022 Oct 1;13(5):1546-1561 ], we have noted some inadvertent errors. The immunofluorescence staining images of Occludin/DAPI and ZO-1/DAPI in the P-gp pcDNA3.1 group were incorrectly presented in Figure 6E due to an oversight during the figure preparation process. We have attached the correct Figure 6, ensuring the accurate depiction of the data.

**Figure 6.**
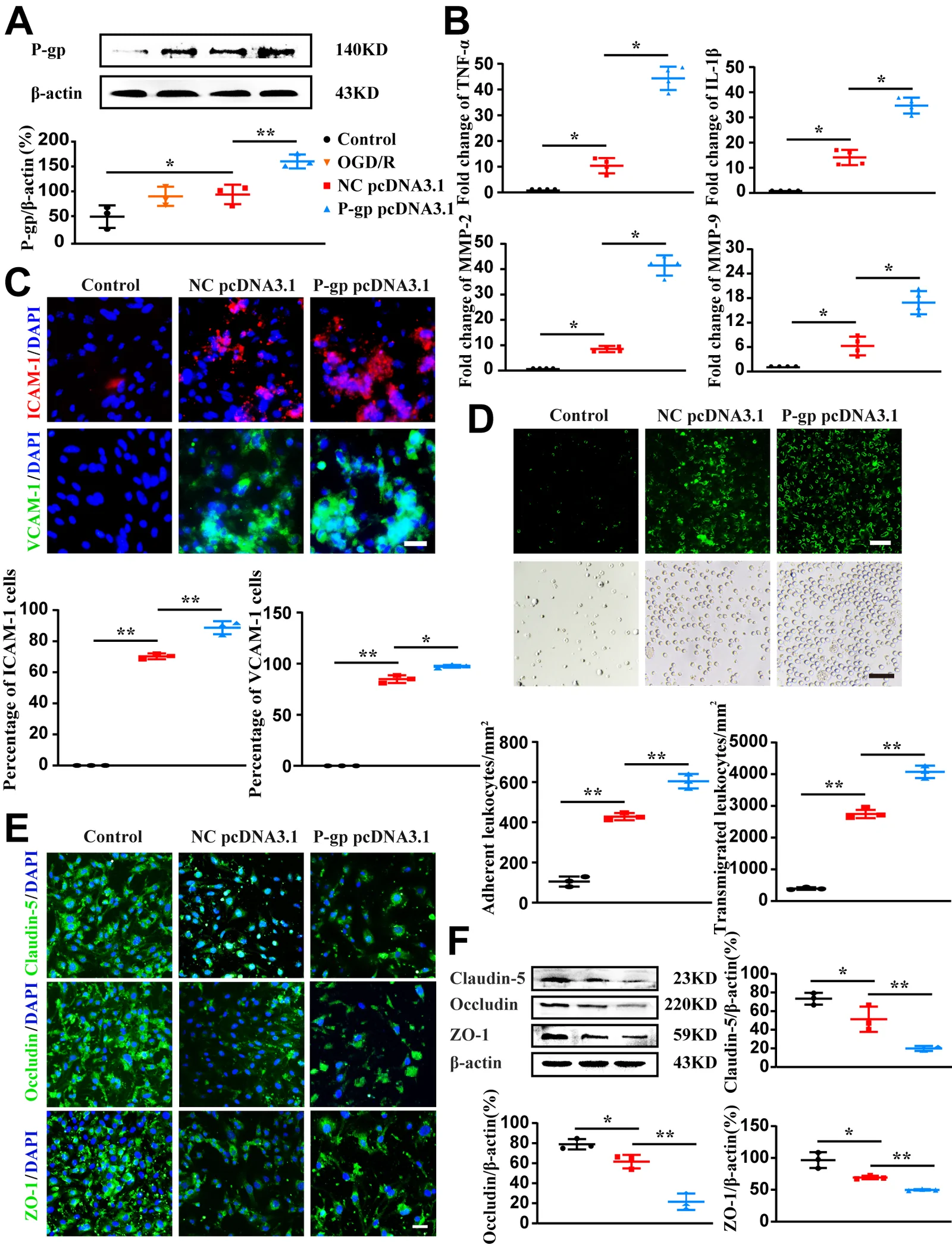
**P-glycoprotein Overexpression Exacerbates Endothelial Dysfunction Following Oxygen Glucose Deprivation/Reoxygenation**. Endothelial cells (bEnd.3) were transfected with P-glycoprotein (P-gp) pcDNA3.1 plasmid, negative control (NC) pcDNA3.1 plasmid, or were untransfected, and then subjected to either oxygen glucose deprivation/reoxygenation (OGD/R) treatment or normal culture conditions. Twenty-four hours thereafter, cells were harvested for immuno-fluorescence staining, adhesion, transendothelial migration, real-time PCR gene expression, and Western blotting analyses. (**A**) Representative Western blotting images and quantification of P-gp protein levels (n = 3). (**B**) Quantification of mRNA levels for TNF-α, IL-1β, MMP-2, and MMP-9 (n = 4). (**C**) Representative immunofluorescence staining images and quantification of ICAM-1 and VCAM-1 expression (n = 3). (**D**) Images and quantification of fluorochrome-labeled leukocyte adhesion to endothelial cells and transendothelial migration (n = 3). (**E**) Representative immunofluorescence staining images for tight junction proteins (Claudin-5, Occludin, and ZO-1). (**F**) Representative Western blotting images and quantification of the expressions of Claudin-5, Occludin, and ZO-1 (n = 3). Scale bars: 40 μm. One-way ANOVA followed by the post hoc Tukey test were used for A, D, E, and F. The Mann-Whitney test was used for B and C. All data are shown as mean ± SD, *P < 0.05, **P < 0.01 between two groups.

We have reviewed all associated data and confirm that these corrections do not affect the conclusions of the study. The scientific conclusions as stated in the original publication remain valid and unchanged. We truly apologize for the errors and the inconvenience caused. We are committed to ensuring the highest standards of scientific integrity and transparency and have taken steps to enhance our figure verification process for future submissions.

