# Supplementary material for: P-Glycoprotein Aggravates Blood Brain Barrier Dysfunction in Experimental Ischemic Stroke by Inhibiting Endothelial Autophagy
Source: Aging Dis. 2022 Oct 1;13(5):1546–61. doi: 10.14336/AD.2022.0225 (PMC9466967; doi:10.14336/AD.2022.0225)
Supplement: Supplementary file 1 [file AD-13-5-1546-s.pdf]

SUPPLEMENTARY DATA

P-Glycoprotein Aggravates Blood Brain Barrier Dysfunction in Experimental Ischemic Stroke by Inhibiting Endothelial Autophagy

Liangliang Huang[1,#], Yan Chen[1,#], Rui Liu[1], Binbin Li[1], Xuan Fei1, Xiang Li[1], Ge Liu[1], Yunman Li[1*], Baohui Xu[2], Weirong Fang[1*]

© 2022. Huang L. et al. Published online at http://www.aginganddisease.org/EN/10.14336/AD.2022.0225

SUPPLEMENTARY DATA

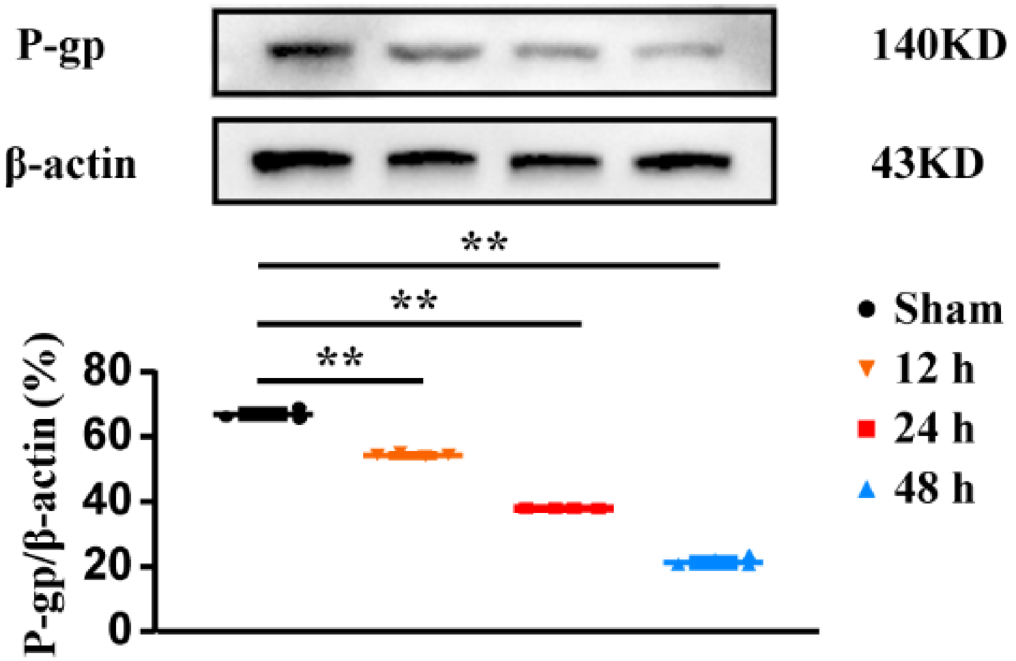

Supplementary Figure 1. Expressions of P-glycoprotein at 12, 24 or 48 h after intracerebroventricular injection. Mice were intracerebroventricularly injected with P-glycoprotein (P-gp) or negative control (NC) siRNA (1.5 μL/10 g body weight) prior to MCAO/R surgery. Twenty-four hours after the surgery, brains were harvested for Western-blotting assay. Western-blotting images and quantification of P-gp levels in brain cortex (n = 4). One-way ANOVA followed by the post hoc Tukey test. All data are mean ± SD, $^{*}P$<0.05, $^{**}P$<0.01 between two groups.

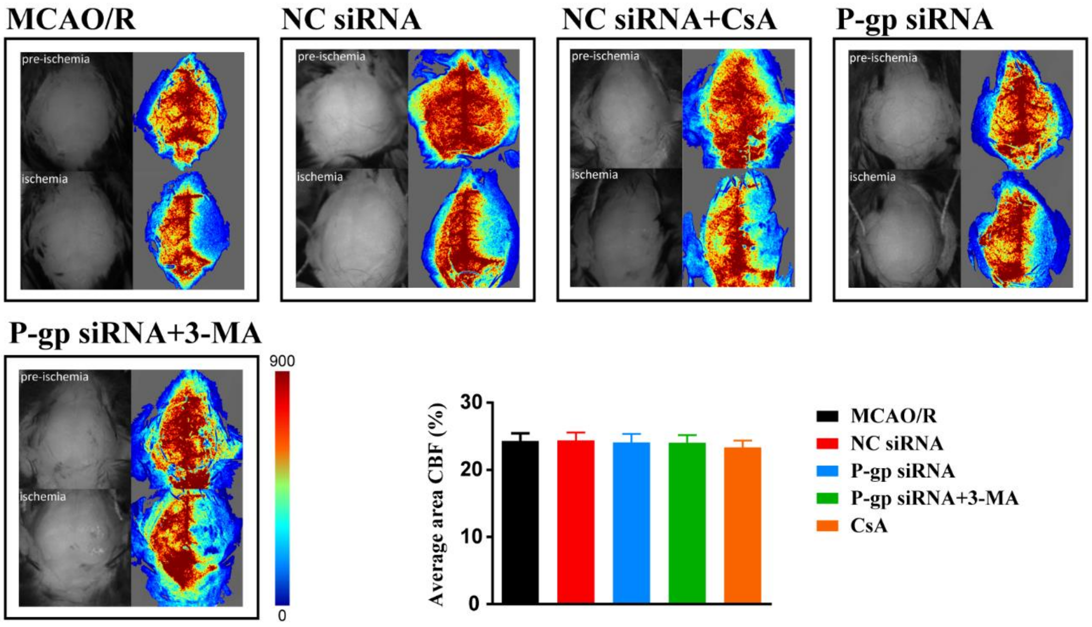

Supplementary Figure 2. Middle cerebral artery occlusion surgery decreased the cerebral blood flow of mice. Representative speckle contrast images and quantification for cerebral blood flow (CBF) before and after middle cerebral artery occlusion surgery in differentially treated mice (n = 6). Mann–Whitney test. All data are shown as mean ± SD, *P*>0.05 between two groups.

© 2022. Huang L. et al. Published online at http://www.aginganddisease.org/EN/10.14336/AD.2022.0225

SUPPLEMENTARY DATA

Supplementary Table 1. The primers of the genes tested.

Target gene	Primer type	Sequence
Mouse TNF-α	Forward	GCACCACCATCAAGGACTCA
	Reverse	TCGAGGCTCCAGTGAATTCG
Mouse IL-1β	Forward	GCCTCGTGCTGTCGGACC
	Reverse	TGTCGTTGCTTGGTTCTCCTTG
Mouse MMP-2	Forward	TCTGGTGCTCCACCACATACAACT
	Reverse	CTGCATTGCCACCCATGGTAAACA
Mouse MMP-9	Forward	GCCCTGGAACTCACACGACA
	Reverse	TTGGAAACTCACACGCCAGAAG
Mouse β-actin	Forward	CATCCGTAAAGACCTTTGCCAAC
	Reverse	ATGGTGCCACCGATCCACA

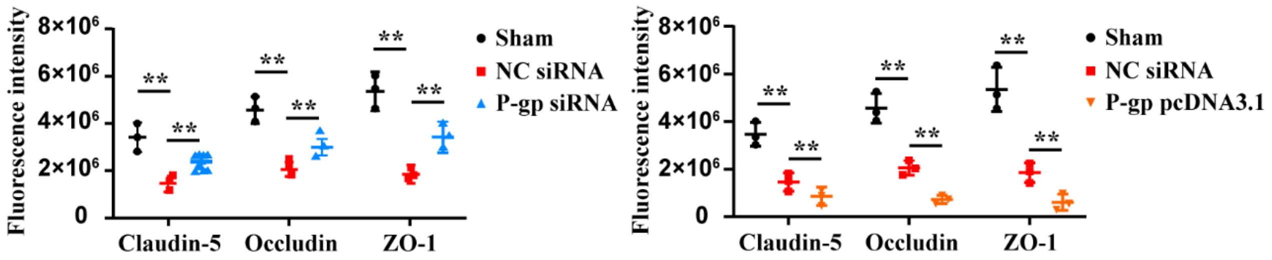

Supplementary Figure 3. P-glycoprotein affects tight junction proteins degradation following endothelial oxygen glucose deprivation/reperfusion. Endothelial cells (bEnd.3) were transfected with P-gp or negative (NC) siRNA, P-gp or NC pcDNA3.1, or un-transfected, and then subjected to either oxygen glucose deprivation/reperfusion (OGD/R) treatment or normal culture conditions. Twenty-four hours thereafter, cells were harvested for immunofluorescence staining. Quantification of tight junction proteins in endothelial cells as representative images showing in figure 5E and 6E (n = 3). Mann–Whitney test. All data are shown as mean ± SD, $^{*}P$<0.05, $^{**}P$<0.01 between two groups.

© 2022. Huang L. et al. Published online at http://www.aginganddisease.org/EN/10.14336/AD.2022.0225
